# Bisphosphonium Amphiphiles Yield Insights into Gram-Negative Bacterial Disinfectant Resistance and Cell Membrane Interactions

**DOI:** 10.1021/acsomega.5c03308

**Published:** 2025-06-05

**Authors:** Elise L. Bezold, Abigail L. E. Young, Carson J. Jaworski, Kevin P. C. Minbiole, Christian A. Sanchez, William M. Wuest

**Affiliations:** † Department of Chemistry, 221304 Emory University, Atlanta, Georgia 30322, United States; ‡ Department of Chemistry and Biochemistry, 1744 Samford University, Birmingham, Alabama 35229, United States; § Department of Chemistry and Biochemistry, 8210 Villanova University, Villanova, Pennsylvania 19085, United States

## Abstract

As microbial resistance to commercially available disinfectants has increased over recent decades, the development of new biocides with distinct mechanisms of action has become a priority. Accordingly, our groups have developed and investigated quaternary phosphonium compounds (QPCs) that have displayed novel mechanisms of bactericidal activity against Gram-negative bacterial species. We aimed to characterize the structure–activity relationship of the cation-separating linker length of diphenylphosphonium scaffolds on membrane interactions and resistance mechanisms using Gram-negative model bacterium Pseudomonas aeruginosa (PAO1 and PA14). Antibacterial activity against lab strain PAO1 and a panel of P. aeruginosa clinical isolates from facilities in Ukraine exhibited potent activity with minimum inhibitory concentrations of 2–4 μM. Surprisingly, the linker length minimally affected the inner-membrane specificity of the QPCs. In comparing the known resistance mechanism for these QPCs, we found that the shorter linker lengths were much more susceptible to efflux by the SmvRA system than the longer linker chain bolaamphiphilic compounds. Additionally, we determined the critical micelle concentration of the QPCs and found that supramolecular aggregation properties do not correlate with the distinct inner-membrane-targeting mechanism of the QPCs. These results represent important advances in the structure-guided investigation of inner-membrane-selective disinfectants.

## Graphical abstract

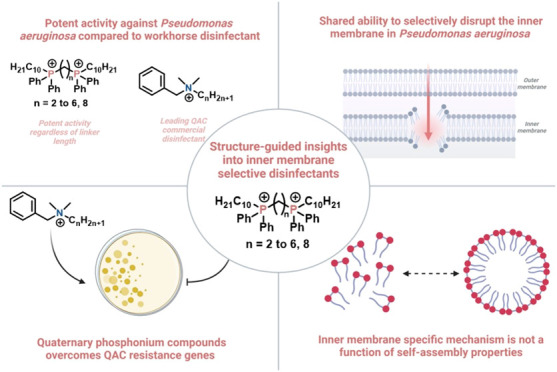

## Introduction

Cationic biocides have served an essential role in infection prevention and control as surface disinfectants, antiseptics, and sanitizers for nearly a century.^1^ These antimicrobial agents are commonly used in domestic, healthcare, food, water, and manufacturing settings.^2^ These biocides utilize a shared mechanism of action, wherein the positively charged head associates with the negatively charged phospholipid cell membrane.^3^ Generally, this association is followed by the insertion of their lipophilic tails into the lipid bilayer, which results in destabilization and ultimately disruption (Figure 1A).

**Figure 1:**
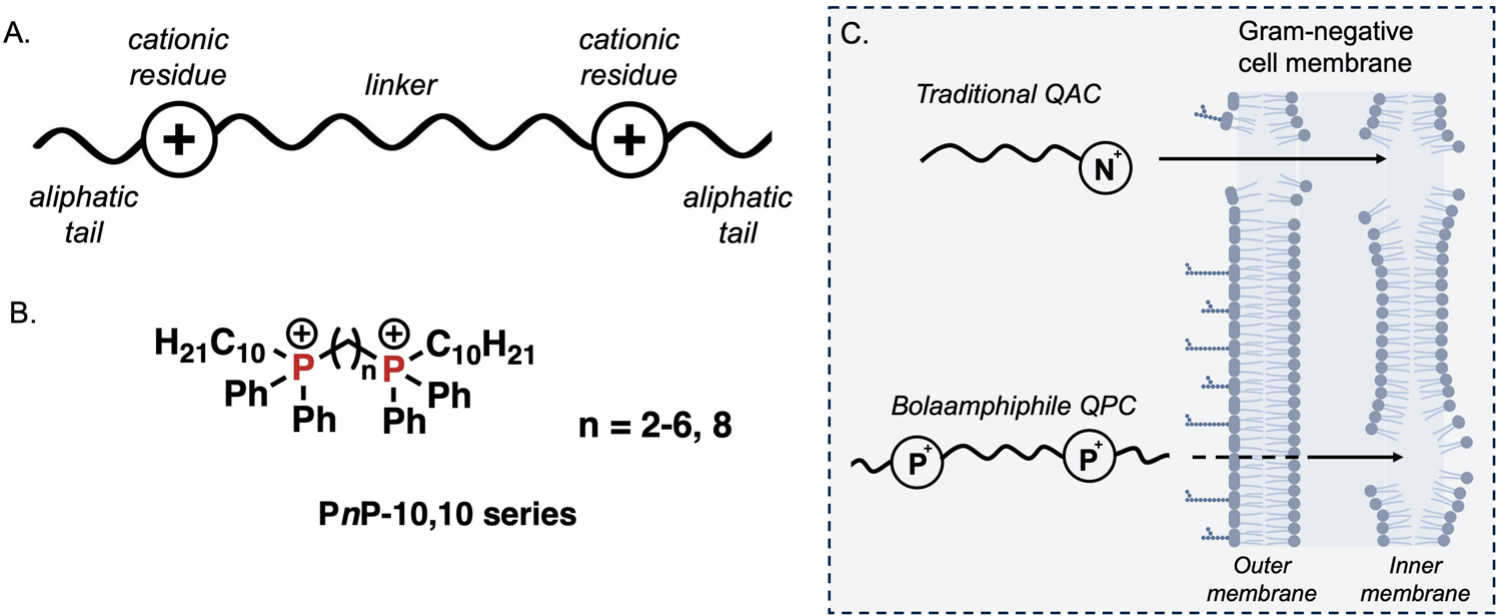
(A) Generalized bolaamphiphile architecture comprising two cationic residues separated by a linker, often featuring aliphatic tails. (B) Bisphosphonium amphiphiles in the present study. (C) Nondiscriminative membrane disruption model of traditional QACs compared to the inner-membrane-selective mechanism of the bolaamphiphile quaternary phosphonium compounds (QPCs) studied.

There exists a wide variety of classes of cationic biocides based on their positively charged element or functional group. Commercially available cationic biocides primarily include quaternary ammonium compounds (QACs) and bisguanides. However, experimental cationic biocide classes additionally encompass sulfonium and phosphonium amphiphiles and significantly vary in both the number of cationic moieties and structural elements among QACs.^4–8^

In recent years, reports of reduced susceptibility to QACs and bisguanides have increased—spurred on by the COVID-19 pandemic and heightened disinfectant usage.^9–11^ Concerningly, the most used QACs, benzalkonium chloride (BAC) and didecyldimethylammonium chloride (DDAC), have been found to be decreasingly effective against high-priority microbial pathogens.^12,13^ This can lead to severe nosocomial outbreaks among vulnerable patients. Due to the importance of these cationic biocides for healthcare settings, this trend is alarming.^14^ Current research highlights the need to develop new disinfectants that can overcome the current and future challenges of surface disinfection.

Cationic biocides with phosphonium moieties have shown great promise as disinfectants. Recent investigations of antimicrobial applications of quaternary phosphonium compounds (QPCs) began with Endo and co-workers in the 1990s.^15,16^ Since then, mono-, bis-, and multiQPCs have all been explored as antimicrobials. In these studies, the bisQPC scaffolds displayed the most potent broad-spectrum activity compared to the mono- and multiQPCs. QPCs with aryl, alkyl, and even phospholium cores have been synthesized, revealing a spectrum of QPCs with potent bioactivity.^17–19^ The Wuest and Minbiole groups have extensively explored novel bisphosphonium amphiphiles.^20–25^ Many of these QPCs possess a bolaamphiphilic architecture, where the two cationic residues are separated by a flexible linker.

Recently, we have shown that a highly effective bisphosphonium bolaamphiphile, P6P-10,10, acts via a unique mechanism of action in Gram-negative bacteria alongside two other nitrogen bolaamphiphiles (octenidine and chlorhexidine) (Figure 1B).^26^ Whereas BAC and DDAC have an indiscriminate mechanism of action, P6P-10,10, OCT, and CHX selectively target the inner membrane of Gram-negative bacteria. These disinfectants warrant considerable attention due to their recently demonstrated high efficacy against clinical isolates of the Gram-negative pathogen Pseudomonas aeruginosa that were tolerant of traditional QACs. In resistance selection and genetic experiments, we discovered that these bolaamphiphiles share the resistance determinant *smvR* in Gram-negative bacteria. The transcriptional regulator SmvR is a TetR-type transcriptional repressor that regulates the expression of *smvA* major facilitator superfamily efflux pump.^27^ Nonetheless, there remained unanswered questions surrounding the structure–activity relationship (SAR) between P6P-10,10 and its bioactivity and bacterial resistance mechanisms (Figure 1C).

Furthermore, we recently examined the effect of linker length on the bioactivity of QAC bolaamphiphiles. Using primarily resorcinol and hydroquinone cores, we explored the effect of the linker length between cationic nitrogen moieties and found that there was no direct effect on minimum inhibitory concentration (MIC) against a panel of seven bacterial strains. The total number of carbons in the side chain appeared to correlate with bioactivity, but the cation spacing seemed immaterial.^28^ In the present study, we sought to further explore how changes in the linker length between the two cationic residues of the bisphosphonium amphiphiles would affect potency and mechanism of action in the model Gram-negative bacterium P. aeruginosa. Furthermore, we examined whether the SmvRA system displayed any substrate preference among compounds with varying linker lengths. Finally, we investigated any connection between the self-assembly behavior, linker length, and mechanism of action. These findings provide important insight into potent bolaamphiphile antimicrobials and bacterial disinfectant resistance.

## Results and Discussion

### P*n*P-10,10 Series Displays Potent Activity against P. aeruginosa

The bisQPC name P6P-10,10 (Figure 1B) reflects two diphenylphosphoniums separated by six methylene groups and two 10-carbon tails extending from each phosphonium. In the present investigation, only changes in the linker length were examined, which varied the total number of carbons. In the initial report,^20^ the 10-carbon tails proved to be the most broadly potent scaffold. Furthermore, traditional bolaamphiphile characteristics are typically a function of linker properties.^29^ By using the P*n*P-10,10 (*n* = 2, 3, 4, 5, 6, 8) series, this allowed for the systematic investigation of this bisphosphonium bolaamphiphile class of antimicrobials.

While five of the bolaamphiphiles (*n* = 2–6) had been previously synthesized, P8P-10,10 was synthesized for the first time, using previously reported procedures.^20^ The bioactivities of our synthesized QPCs were determined via minimum inhibitory concentration (MIC) and red blood cell (RBC) hemolysis (lysis_20_) assays. The hemolysis assay was used as a proxy for cytotoxicity. Benzalkonium chloride (BAC) served as the control compound. To determine MIC values, the compounds were screened against two P. aeruginosa reference strains PAO1 and PA14.

All six QPCs displayed potent inhibitory activity against both strains of P. aeruginosa (Table 1) with the longer linker length (*n* = 4–8) exhibiting modestly better bioactivity (MIC of 2 μM) than the shorter linker length compounds (MIC of 4 μM). This is in stark contrast to much higher MIC of BAC at 250 and 125 μM for PAO1 and PA14, respectively. The difference in MIC reveals the advantage of cationic biocides with the inner-membrane-specific mechanism of action, in contrast to the indiscriminate membrane disruption mechanism of BAC. Most importantly, the low MICs of these QPCs represent the promise of this class of bolaamphiphiles in inhibiting hard-to-kill Gram-negative pathogens.

**Table 1:** MICs and Lysis_20_ Data of the P*n*P-10,10 Series against P. aeruginosa Laboratory Strains

	minimum inhibitory concentration (μM)		
compound	PAO1	PA14	lysis_20_
P2P-10,10	4	4	8
P3P-10,10	4	4	8
P4P-10,10	2	2	4
P5P-10,10	2	2	4
P6P-10,10	2	2	4
P8P-10,10	2	2	4
BAC	250	125	63

In terms of the lysis of red blood cells, which we used as a proxy for eukaryotic toxicity, we found that our panel of QPCs exhibited significant toxicity with lysis_20_ values of 4 or 8 μM, whereas BAC displayed a lysis_20_ value of 63 μM.

We further wanted to study the effect of the linker length on bioactivity against P. aeruginosa clinical isolates provided by Walter Reed Army Institute of Research from facilities in Ukraine. Previous reports have demonstrated the impact of hospital-acquired infections from antibiotic-resistant bacteria, which is ongoing in the present wartime period there.^30,31^ We sought to evaluate the efficacy of our bisphosphonium compounds on clinical isolates from military settings with known disinfectant resistance genes. Eight clinical isolates with varying genetic backgrounds were tested (Table 2). These strains harbored genes related to disinfectant resistance, specifically *qacE* and/or *qacE*Δ genes. The *qacE* gene is implicated in the efflux of antiseptics and intercalating dyes, whereas *qacE*Δ is described as a semifunctional version of the *qacE* gene. However, the role the gene plays in QAC resistance may be limited.^32,33^ Since these clinical isolates harbor resistance genes to QACs, we hypothesized that our library of QPCs would exhibit superior efficacy compared to the control compound, BAC, with our *n* = 4–8 linker QPCs showing greater bioactivity.

**Table 2:** MICs of the P*n*P-10,10 Series against P. aeruginosa Clinical Isolates with *qacE*Δ (122268, 740699, 740761, 122323, 122372, 122357, 567268), *qacE* (122372), and No *qac* Family Gene (821282)

	minimum inhibitory concentration (μM)							
	122268	740699	740761	122323	122357	567268	122372	821382
compound	qac*E*Δ	qac*E*Δ	qac*E*Δ	qac*E*Δ	qac*E*Δ	qac*E*Δ	qac*E*, qac*E*Δ	no qac gene
P2P-10,10	4	4	4	4	4	4	4	4
P3P-10,10	4	4	4	4	4	4	4	4
P4P-10,10	2	2	2	2	2	2	2	2
P5P-10,10	2	2	2	2	2	2	2	2
P6P-10,10	2	2	2	2	2	2	2	2
P8P-10,10	2	4	2	2	2	2	2	2
BAC	>250	>250	>250	125	125	125	125	125

We observed that across all eight strains, P2P-10,10 and P3P-10,10 showed lower bioactivity than the *n* = 4–8 QPCs. This modestly lower activity of the shorter chain bolaamphiphiles was observed for the laboratory strains as well. It appears that the presence or absence of *qacE* and *qacE*Δ had no effect on bolaamphiphile bioactivity in the strains tested. Ultimately, all QPCs exhibited superior bioactivity compared to commercial disinfectant BAC (MIC ≥ 125 μM), which demonstrates the promise of this class of compounds.

### SmvRA System Is Specific to Shorter Linker Length Substrates

We then screened our library of compounds against our previously reported P6P-10,10-resistant mutant of PAO1 (PAO1-P6P^R^) and the *smvR*-complementation strain (PAO1-P6P^R^ + *smvR*). We hypothesized that cross-resistance would be observed between our PAO1-P6P^R^ strain and the library of compounds synthesized, based on their structural similarity.

Cross-resistance was observed most distinctly in P2P-10,10, P3P-10,10, P4P-10,10, and P5P-10,10, where as high as 8-fold increases in MIC were reported (Table 3). Interestingly, greater resistance is observed for compounds with shorter linker lengths, such as P2P-10,10, than the change in MIC observed for P6P-10,10. This suggests that the SmvRA system may preferentially efflux shorter linker length amphiphiles over the longer linker length bolaamphiphiles. Further supporting this hypothesis, no change in the MIC was observed between PAO1 and the P6P-resistant mutant for P8P-10,10. This result is promising and could hint at longer linkers having a different resistance determinant than compounds with linkers of six carbons or less.

**Table 3:** MICs of the P*n*P-10,10 Series against P. aeruginosa PAO1, P6P-10,10-Resistant PAO1, and P6P-10,10-Resistant PAO1 with *smvR* Complement

compound	PAO1	PAO1-P6P^R^	PAO1-P6P^R^ + smvR
P2P-10,10	4	32	16
P3P-10,10	4	16	16
P4P-10,10	2	16	8
P5P-10,10	2	16	8
P6P-10,10	2	8	4
P8P-10,10	2	2	2
BAC	250	125	125

### P*n*P-10,10 Series Share Common Mechanism of Action

We next sought to characterize the interactions between the P*n*P-10,10 bolaamphiphile disinfectants and the cell membranes of P. aeruginosa. While common disinfectants like BAC and DDAC share a nondiscriminative membrane disruption mechanism, P6P-10,10 alongside biguanides octenidine and chlorhexidine are inner-membrane-selective. In light of this discovery, SAR questions concerning structural features such as the linker length remain. A previous study suggests that the linker length influences the mechanism by which bolaamphiphiles interact with the membrane.^34^ We hypothesized that the short linker length QPCs (e.g., P2P-10,10) would demonstrate differing mechanisms when compared to the longer linker length QPCs (e.g., P8P-10,10).

As a proxy for inner-membrane disruption, we utilized a 3,3′-dipropylthiadicarbocyanine iodide [DiSC_3_-(5)] membrane depolarization procedure. The fluorescent dye DiSC_3_-(5) binds to the bacterial cytoplasmic membrane when there is a robust cell membrane potential. Upon membrane potential dissipation, a characteristic event in disinfectant-induced membrane disruption, DiSC_3_-(5) is released from the cell surface and can be detected by using fluorescence spectroscopy.

We observed the same degree of membrane depolarization with each P*n*P-10,10 compound (Figure 2A). Additionally, these depolarization events occurred within a comparable time-course. These results confirmed a shared ability to disrupt the inner membrane of P. aeruginosa PAO1, but no clear SAR trend could be deduced.

**Figure 2:**
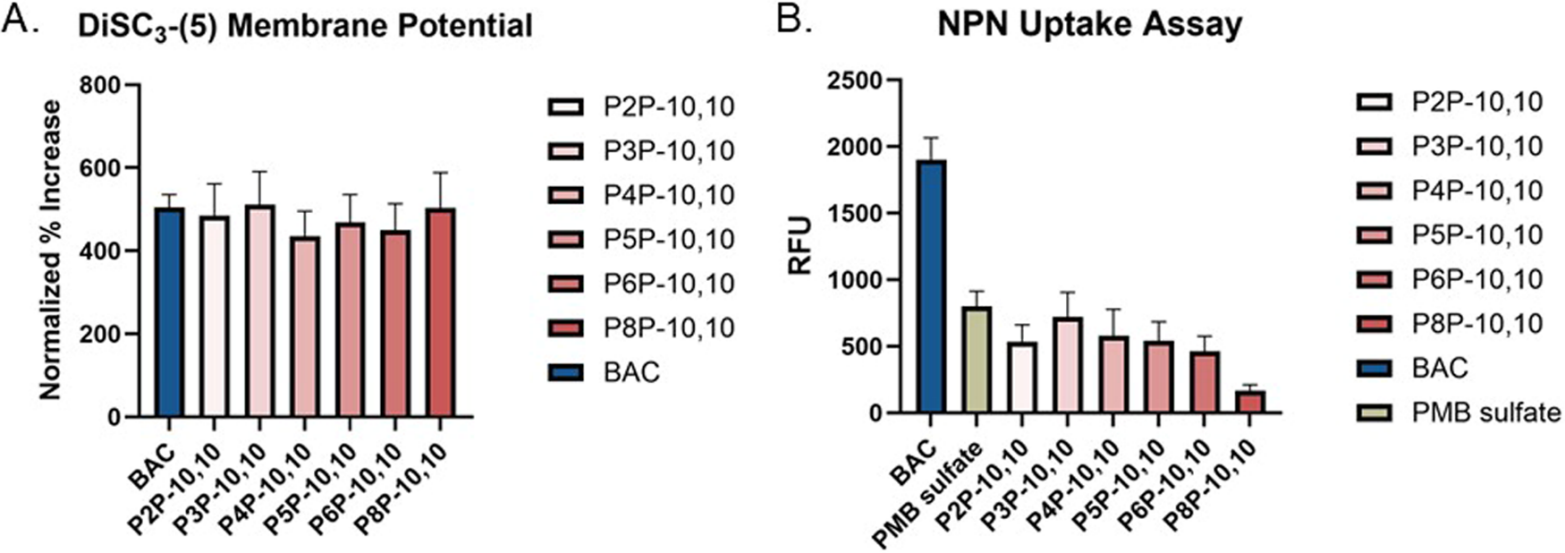
(A) DISC_3_-(5) results in PAO1 show similar levels of inner-membrane disruption resulting in depolarization among all compounds tested after 60 min. (B) *N*-Phenyl-1-naphthylamine (NPN) uptake in PAO1 reveals minimal outer membrane disruption for the P*n*P-10,10 series in contrast to BAC after 60 min. P*n*P-10,10 compounds were tested at 5 μM for both experiments, while BAC was tested at 125 μM and polymyxin (PMB) was tested at 0.125 μM.

Furthermore, we performed a *N*-phenyl-1-naphthylamine (NPN) uptake assay to examine the outer membrane disruption. NPN is a cell membrane impermeable fluorescent dye. Upon permeabilization of the outer membrane, NPN intercalates into the lipid bilayer and can be detected by using fluorescence spectroscopy. Our results revealed less uptake of NPN induced in all P*n*P-10,10 treatments compared to the commercial QAC BAC (Figure 2B).

Comparing the P*n*P-10,10 treatments, there were minor differences. Some lower disruption was observed with P2P-10,10 and P8P-10,10. However, this apparent parabolic trend in NPN uptake peaked at P3P-10,10. The nonlinear relationship may indicate that other interactions are occurring, which play a role in the selective permeability of QPCs in PAO1. Out of the six compounds tested, P8P-10,10 treatment resulted in the least outer membrane disruption, potentially due to its bolaamphiphilic nature.

### Critical Micelle Concentrations (CMCs) Do Not Correlate with the Inner-Membrane-Specific Mechanism

To explore additional possible factors influencing the mechanism of action, we investigated the supramolecular aggregation properties of P*n*P-10,10 by measuring the critical micelle concentration (CMC). CMC is the minimum concentration at which the monomeric forms of the surfactant self-assemble into aggregates or micelles.^35^ CMC mainly depends on the hydrophobicity of the surfactant and can be strongly influenced by the presence of salts.^36^ Furthermore, supramolecular assembly can affect biological activity, such as toxicity. Toxicity and self-assembly have been shown to be closely correlated.^37–39^ It has been shown that dynamic surface tension properties of disinfectants correlate with suspension time-kill efficacy but not with MICs.^40^ We hypothesized that the related physical property of micelle formation may influence surfactant–membrane interactions.

To obtain CMC values for our P*n*P-10,10 series, we adapted a fluorescence-based pyrene assay to determine the self-assembly point for each compound (Figure 3).^41^ Pyrene has unique fluorescence emissions when it is in a free aqueous state and when it is in a bound micelle state. These unique fluorescence profiles can be compared ratiometrically by analyzing the spectral ratio (SR) of fluorescence emissions of the solution. As the concentration of micelles increases, the concentration of free pyrene decreases as they become bound to the micelle. This results in an increase in the value of the ratio of the two emission bands (383/372 nm). By adapting this procedure to a 96-well plate format, we were able to determine the CMCs using the pyrene method with only several milligrams of analyte.

**Figure 3:**
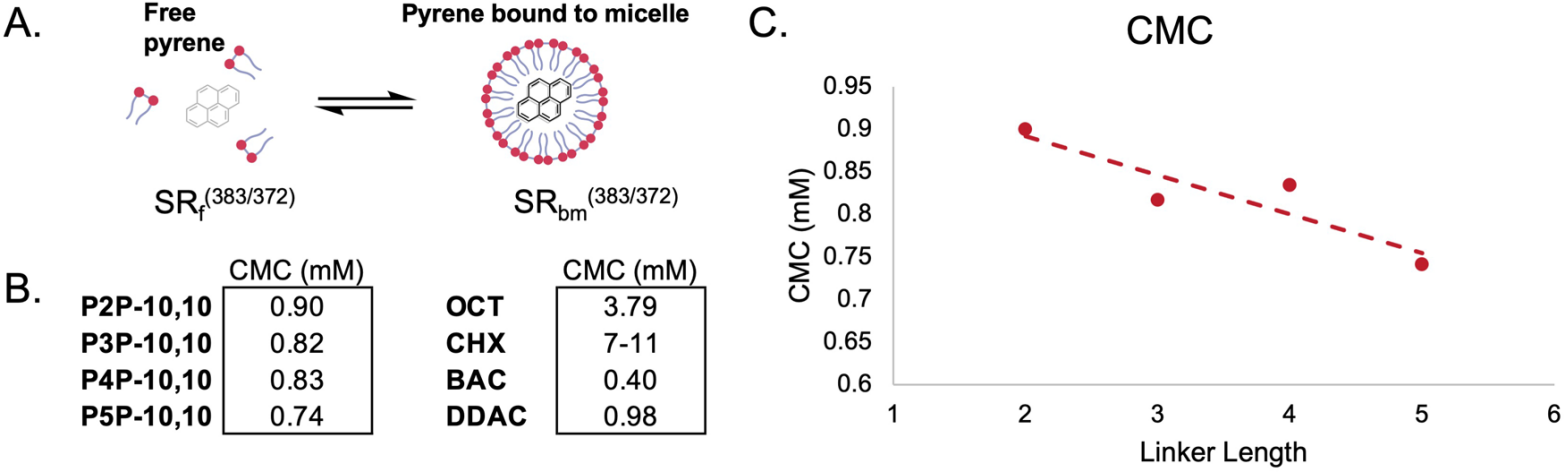
(A) Representation of the ratiometric pyrene fluorescence assay used to determine the CMC. (B) CMCs of P*n*P-10,10 series alongside literature values for OCT,^41^ CHX,^42^ BAC,^43^ and DDAC.^44^ (C) Trendline of CMC values as a function of the linker length (*R* ^2^ = 0.83).

Only linker lengths with fewer than six methylenes were soluble in the aqueous pyrene solution at appropriate concentrations for testing. The four P*n*P-10,10 surfactants suitable for testing had CMC values below 1 mM. These CMCs were most similar to the CMCs of BAC and DDAC at 0.40 and 0.98 mM, respectively. In contrast, the other inner-membrane-specific bolaamphiphiles, OCT and CHX, had significantly higher CMC values. From these data, we conclude that the inner-membrane-specific mechanism is not a function of self-assembly properties.

Finally, we note that as the linker length between the two cationic residues increases in the P*n*P-10,10 series, there is a decrease in the CMC value. This trend accords well with a study done in histidyl bolaamphiphiles, where the increasing linker length decreased the critical aggregation concentration.^45^ Examining the initial report of the P*n*P-10,10 series, there did not appear to be any difference in hemolytic properties.^20^ This suggests that either the difference in CMC may not be enough to appreciably affect toxicity or using hemolysis as a proxy for toxicity may not reflect potential variations in activity.

## Conclusions

In summary, we have investigated how the linker length affects bisQPC bolaamphiphiles in terms of inhibitory potency, efflux by the SmvRA system, mechanism of action, and supramolecular aggregation. The P*n*P-10,10 series represents a highly effective class of quaternary phosphonium disinfectants with single-digit micromolar inhibitory activity against the Gram-negative pathogen P. aeruginosa where the shorter linker length (*n* = 2–3) exhibited modestly lower bioactivity. We found that the SmvRA system has a preference for shorter linker length biscationic structures, though more work should be done to elucidate the breadth of its substrate scope. This class of phosphonium bolaamphiphiles appears to share a common mechanism of action in which the outer membrane of the bacteria stays intact, while the inner membrane is disrupted. Despite variation in the linker length, little difference in the outer and inner-membrane perturbation was observed. These results suggest that other structural characteristics like the alkyl tail or phosphonium substituent may play a larger role in affecting the mechanism of action. Finally, investigations into the self-assembly properties of the surfactant were undertaken. By comparing the CMCs of the P*n*P-10,10 series to other commercial QACs and bolaamphiphiles, we do not observe any noticeable correlation between the mechanism of action and micelle formation.

Although much remains to be explored in elucidating the structural properties that are responsible for the inner-membrane-specific mechanism of action, this study lends important insight into how novel antimicrobials can be designed to evade bacterial resistance and how supramolecular assembly might influence the mechanism of action.

## Methods

### Chemicals

Nonaqueous reactions were carried out under an atmosphere of argon in flame-dried glassware. *N*,*N*-Dimethylformamide (DMF) was dried by passage through alumina. All commercial reagents and anhydrous solvents were used and received (from Sigma-Aldrich, Oakwood Chemical, Combi Blocks, Alfa Aesar, Fisher Scientific, TCI America, or AK Scientific) without further purification. Reactions were monitored by analytical thin-layer chromatography (TLC) using EMD Millipore silica gel 60 F_254_ precoated plates and visualized using UV and/or vanillin or KMnO_4_ stains. Nuclear magnetic resonance spectra were recorded using either a Bruker 400 (400/101 MHz) or a Varian INOVA 400 (400/101 MHz). Chemical shifts are reported in parts per million (ppm) relative to tetramethylsilane and are referenced to the residual solvent signal. Signal patterns are indicated as follows: singlet (s), doublet (d), triplet (t), quartet (q), multiplet (m), and broad signal (br). High-resolution mass spectra were recorded on a ThermoScientific Exactive Plus Orbitrap MS.

### Synthesis of P8P-10,10

To 1,8-bis­(diphenylphosphino)­octane (0.100 g, 0.207 mmol, 1 equiv) were added 1-bromodecane (109 μL, 0.117 g, 0.518 mmol, 2.5 equiv) and DMF (3 mL). The solution was heated to 100 °C and stirred for 24 h. After the mixture was cooled to room temperature, the contents of the reaction flask were concentrated using rotary evaporation. The resulting oil was triturated with 1:1 ether/hexanes (20 mL) and cooled at −25 °C overnight. The trituration solvent was discarded, and the resulting precipitate was dissolved in dichloromethane (5 mL). The solution was transferred to a clean vial and concentrated using rotary evaporation to afford P8P-10,10 as a white crystalline powder (0.168 mg, 88%). ^1^H NMR (400 MHz, CDCl_3_) δ 7.91 (dd, *J* = 12.0, 7.7 Hz, 8H), 7.77 (dd, *J* = 8.3, 6.4 Hz, 4H), 7.69 (td, *J* = 7.6, 3.1 Hz, 8H), 3.31 (d, *J* = 13.6 Hz, 8H), 1.48 (s, 16H), 1.31–1.22 (m, 11H), 1.18 (s, 17H), 0.85 (t, *J* = 6.9 Hz, 6H). ^13^C NMR (101 MHz, CDCl_3_) δ 134.74, 134.71, 133.44, 133.34, 130.48, 130.36, 118.54, 117.72, 77.36, 31.93, 30.64, 30.49, 29.80, 29.64, 29.55, 29.35, 29.20, 27.73, 22.75, 22.37, 22.31, 22.10, 21.89, 21.75, 21.70, 21.61, 14.22. ^31^P NMR (162 MHz, CDCl_3_) δ 28.58. High-resolution mass spectrometry (HRMS) accurate mass (ESI+): found 382.7773, C_52_H_78_P_2_[−2Br−]^2+^ requires 382.2789.

### Bacterial Strains and Growth Conditions

P. aeruginosa PAO1 was acquired from Prof. Bettina A. Buttaro (Lewis Katz School of Medicine, Temple University). MRSN 122268, 740699, 740761, 122323, 122357, 567268, 122372, and 821382 are clinical isolates of P. aeruginosa obtained from the Multidrug resistant Organism Repository and Surveillance Network (MRSN) provided by the Department of Defense. Bacterial strains were streaked onto lysogeny broth (LB) agar plates and incubated at 37 °C overnight. Single colonies were used to inoculate liquid cultures and incubated for 18 h at 37 °C with shaking.

### Minimum Inhibitory Concentration (MIC) Assays

To determine the MIC values, compounds were serially diluted 2-fold from stock solutions (1.0 mM) to yield 12 100-μL test concentrations, wherein the starting concentration of dimethyl sulfoxide (DMSO) was 2.5%. Overnight cultures of each strain were diluted to ca. 10^6^ CFU/mL in Mueller–Hinton broth (MHB) and regrown to midexponential phase, as determined by optical density recorded at 600 nm (OD_600_). All cultures were then diluted again to ca. 10^6^ CFU/mL and 100 μL were inoculated into each well of a U-bottom 96-well plate (Falcon, 351177) containing 100 μL of compound solution. Plates were incubated statically at 37 °C for 72 h, upon which wells were evaluated visually for bacterial growth. The MIC was determined as the lowest concentration of compound resulting in no bacterial growth visible to the naked eye, based on the highest value in three independent experiments. Aqueous DMSO controls were conducted for each strain. All P. aeruginosa strains were grown with shaking at 37 °C overnight from single colonies in 5 mL of BD Mueller–Hinton broth (MHB).

### Red Blood Cell (RBC) Lysis Assay (Lysis_20_)

RBC lysis assays were performed on mechanically defibrinated sheep blood (Hemostat Laboratories: DSB030). An aliquot of 1.5 mL blood was placed into a microcentrifuge tube and centrifuged at 10,000 rpm for 10 min. The supernatant was removed, and the cells were resuspended with 1 mL of phosphate-buffered saline (PBS). The suspension was centrifuged as described above, the supernatant was removed, and cells were resuspended 4 additional times in 1 mL of PBS. The final cell suspension was diluted 20-fold with PBS. Compounds were serially diluted with PBS 2-fold from stock solutions (1.0 mM) to yield 100 μL of 12 test concentrations on a U-bottom 96-well plate (Falcon, 351177), wherein the starting concentration of DMSO was 2.5%. To each of the wells, 100 μL of the 20-fold suspension dilution was then inoculated. The concentration of DMSO in the first well was 2.5%, resulting in DMSO-induced lysis at all concentrations >63 μM. TritonX (1% by volume) served as a positive control (100% lysis marker) and sterile PBS served as a negative control (0% lysis marker). Samples were then placed in an incubator at 37 °C and shaken at 200 rpm. After 1 h, the samples were centrifuged at 2000 rpm for 10 min. 100 μL of the supernatant was transferred to a fresh flat-bottom 96-well plate (Corning, 351172), and the absorbance of the supernatant was measured with a UV spectrometer at a 540 nm wavelength. The concentration inducing 20% RBC lysis was then calculated for each compound based upon the absorbances of the TritonX and PBS controls. Aqueous DMSO controls were conducted as appropriate for each compound.

### NPN Uptake Assay

P. aeruginosa PAO1 were grown overnight in LB and then regrown from a 1:100 dilution in fresh media for 5 h to an OD_600_ of 0.500. Cells were harvested by centrifugation (4000 rpm, 25 °C, 10 min), washed twice with an assay buffer (5 mM *N*-(2-hydroxyethyl)­piperazine-*N*′-ethanesulfonic acid [HEPES], 5 mM glucose, pH 7.2), and resuspended in the assay buffer to a final OD_600_ of 1. Then, 100 μL of washed cells and 100 μL of the assay buffer containing 20 μM NPN were mixed together and incubated for 10–30 min. 198 μL of cells and NPN were added to a 96-well optical-bottom black plate. Either 2 μL of a chemical compound or the corresponding solvent was added to each well, and fluorescence was immediately monitored at an excitation wavelength of 350 nm and an emission wavelength of 420 nm for 7 min at 30 s intervals

$$F_{\mathrm{obs}}=\mathrm{NPN}+\mathrm{cells}+\mathrm{compound}$$

$$F_{\mathrm{control}}=\mathrm{NPN}+\mathrm{cells}$$

$$F_{b}=\mathrm{NPN}$$

$$\mathrm{NPN uptake}=\left(F_{\mathrm{obs}}-F_{b}\right)-\left(F_{\mathrm{control}}-F_{b}\right)$$

20 μM NPN in assay buffer was made from a 5 mM stock of NPN in acetone.

### DISC_3_-(5) Depolarization Assay

P. aeruginosa PAO1 were grown overnight in LB and then regrown from a 1:100 dilution in fresh media. Mid-log phase bacteria (OD_600_ = 0.4–0.6) were harvested, washed once, and resuspended in HEPES buffer (5 mM HEPES at pH 7.2) to an optical absorbance of OD_600_ = 0.05. Then, 100 μL of 10 mM ethylenediaminetetraacetic acid (EDTA) was added to 5 mL of resuspended cells for a final concentration of 200 μM EDTA. The bacterial solution was then gently mixed and then let sit for 2 min. Afterward, 5 μL of 0.75 mM DISC_3_-(5) was added to the solution for a final concentration of 0.75 μM. Following another gentle mix, the solution was left to incubate in the dark at 37 °C. After incubation, 125 μL of 4 M KCl was added to the cells for a final concentration of 100 mM KCl. Finally, 198 μL of cells and DISC_3_-(5) were added to a 96-well optical-bottom black plate. Either 2 μL of a chemical compound, or the corresponding solvent, was added to each well. The excitation wavelength was 622 nm, and the emission wavelength was 670 nm. The release of DISC_3_-(5) was measured by the increase in the fluorescence of DISC_3_-(5) for 60 min as a measure of inner-membrane depolarization.

### Pyrene CMC Determination Assay

10.1 mg of pyrene and 80 mL of ethanol were added into a 100 mL volumetric flask. After the mixture was shaken well until fully dissolved, ethanol was filled to the mark to prepare a 500 μM solution. A 1:1000 dilution of the pyrene solution in water was performed to prepare a 0.5 μM solution of pyrene. A 1.2 mM stock solution of each P­(*n*)­P-10,10 compound was prepared with pyrene solution. This was the test solution used for fluorescence experiments, containing both 0.5 μM pyrene and 1.2 mM surfactant.

The test solution was further diluted in a 96-well plate with additional 0.5 μM pyrene solution to obtain surfactant concentrations between 0.5 and 1.2 mM while maintaining 0.5 μM of pyrene. The final volume in each well was 200 μL. Exciting at 336 nm, the fluorescence emission of pyrene at 374 (*I*_1_) and 382 nm (*I*_3_) was recorded. The *I*_1_/*I*_3_ intensity ratio was plotted against the concentration of each sample. The CMC value was determined from the intersection of the best-fit lines, corresponding to the minimum surfactant concentration required for the formation of stable micelles in aqueous solution.

## Supplementary Material



## Notes

Detailed synthetic procedures for the described compounds, ^1^H and ^13^C NMR data and spectra, data on strains and growth conditions, detailed biological assays, and pyrene critical micelle determination plots for the reported compounds (PDF)

## References

[1] Maillard J.-Y. (2005). Antimicrobial
Biocides in the Healthcare Environment: Efficacy, Usage, Policies,
and Perceived Problems. Ther. Clin. Risk Manage..

[2] Beatriz M.
P. P., Ilias T. (2019). Benzalkonium
Chlorides: Uses, Regulatory Status, and
Microbial Resistance. Appl. Environ. Microbiol..

[3] Gilbert P., Moore L. E. (2005). Cationic Antiseptics:
Diversity of Action under a Common
Epithet. J. Appl. Microbiol..

[4] Feliciano J. A., Leitgeb A. J., Schrank C. L., Allen R. A., Minbiole K. P. C., Wuest W. M., Carden R. G. (2021). Trivalent
Sulfonium Compounds (TSCs):
Tetrahydrothiophene-Based Amphiphiles Exhibit Similar Antimicrobial
Activity to Analogous Ammonium-Based Amphiphiles. Bioorg. Med. Chem. Lett..

[5] Hirayama M. (2012). The Antimicrobial
Activity, Toxicity and Antimicrobial Mechanism of a New Type of Tris­(Alkylphenyl)­Sulfonium. Biocontrol Sci..

[6] O’Toole G. A., Wathier M., Zegans M. E., Shanks R. M. Q., Kowalski R., Grinstaff M. W. (2012). Diphosphonium
Ionic Liquids as Broad-Spectrum Antimicrobial
Agents. Cornea.

[7] Terekhova N. V., Khailova L. S., Rokitskaya T. I., Nazarov P. A., Islamov D. R., Usachev K. S., Tatarinov D. A., Mironov V. F., Kotova E. A., Antonenko Y. N. (2021). Trialkyl­(Vinyl)­Phosphonium
Chlorophenol Derivatives
as Potent Mitochondrial Uncouplers and Antibacterial Agents. ACS Omega.

[8] McDonough D., Sanchez C. A., Wuest W. M., Minbiole K. P. C. (2025). Recent Developments
in Antimicrobial Small Molecule Quaternary Phosphonium Compounds (QPCs)
– Synthesis and Biological Insights. RSC Med. Chem..

[9] Maillard J.-Y. (2022). Impact
of Benzalkonium Chloride, Benzethonium Chloride and Chloroxylenol
on Bacterial Antimicrobial Resistance. J. Appl.
Microbiol..

[10] Russell A. D. (1999). Bacterial
Resistance to Disinfectants: Present Knowledge and Future Problems. J. Hosp. Infect..

[11] Mahoney A. R., Safaee M. M., Wuest W. M., Furst A. L. (2021). The Silent
Pandemic:
Emergent Antibiotic Resistances Following the Global Response to SARS-CoV-2. iScience.

[12] Maillard J.-Y., Pascoe M. (2024). Disinfectants and Antiseptics: Mechanisms
of Action
and Resistance. Nat. Rev. Microbiol..

[13] Boyce J. M. (2023). Quaternary
Ammonium Disinfectants and Antiseptics: Tolerance, Resistance and
Potential Impact on Antibiotic Resistance. Antimicrob.
Resist. Infect. Control.

[14] Hanczvikkel A., Tóth Á., Kopcsóné Németh I.
A., Bazsó O., Závorszky L., Buzgó L., Lesinszki V., Göbhardter D., Ungvári E., Damjanova I., Erőss A., Hajdu Á. (2024). Nosocomial Outbreak
Caused by Disinfectant-Resistant Serratia Marcescens in an Adult Intensive
Care Unit, Hungary, February to March 2022. Eurosurveillance.

[15] Kanazawa A., Ikeda T., Endo T. (1993). Polymeric
Phosphonium Salts as a
Novel Class of Cationic Biocides. III. Immobilization of Phosphonium
Salts by Surface Photografting and Antibacterial Activity of the Surface-Treated
Polymer Films. J. Polym. Sci., Part A: Polym.
Chem..

[16] Kanazawa A., Ikeda T., Endo T. (1994). Synthesis and Antimicrobial
Activity
of Dimethyl- and Trimethyl-Substituted Phosphonium Salts with Alkyl
Chains of Various Lengths. Antimicrob. Agents
Chemother..

[17] Kodjo
Amengor C. D., Amaning Danquah C., Adusei E. B. A., Kekessie F. K., Ofosu-Koranteng F., Peprah P., Harley B. K., Orman E., Adu J., Saaka Y. (2022). Synthesized Phosphonium Compounds Demonstrate Resistant
Modulatory and Antibiofilm Formation Activities against Some Pathogenic
Bacteria. Heteroat. Chem..

[18] Ermolaev V. V., Arkhipova D. M., Miluykov V. A., Lyubina A. P., Amerhanova S. K., Kulik N. V., Voloshina A. D., Ananikov V. P. (2022). Sterically Hindered
Quaternary Phosphonium Salts (QPSs): Antimicrobial Activity and Hemolytic
and Cytotoxic Properties. Int. J. Mol. Sci..

[19] Lukáč M., Devínsky F., Pisárčik M., Papapetropoulou A., Bukovský M., Horváth B. (2017). Novel Phospholium-Type Cationic Surfactants:
Synthesis, Aggregation Properties and Antimicrobial Activity. J. Surfactants Deterg..

[20] Sommers K. J., Michaud M. E., Hogue C. E., Scharnow A. M., Amoo L. E., Petersen A. A., Carden R. G., Minbiole K. P. C., Wuest W. M. (2022). Quaternary
Phosphonium Compounds: An Examination of Non-Nitrogenous Cationic
Amphiphiles That Evade Disinfectant Resistance. ACS Infect. Dis..

[21] Spahr A. C., Michaud M. E., Amoo L. E., Sanchez C. A., Hogue C. E., Thierer L. M., Gau M. R., Wuest W. M., Minbiole K. P. C. (2022). Rigidity-Activity
Relationships of BisQPC Scaffolds against Pathogenic Bacteria. ChemMedChem.

[22] Brayton S. R., Toles Z. E. A., Sanchez C. A., Michaud M. E., Thierer L. M., Keller T. M., Risener C. J., Quave C. L., Wuest W. M., Minbiole K. P. C. (2023). Soft QPCs: Biscationic
Quaternary Phosphonium Compounds
as Soft Antimicrobial Agents. ACS Infect. Dis..

[23] Thierer L. M., Petersen A. A., Michaud M. E., Sanchez C. A., Brayton S. R., Wuest W. M., Minbiole K. P. C. (2023). Atom Economical QPCs: Phenyl-Free
Biscationic Quaternary Phosphonium Compounds as Potent Disinfectants. ACS Infect. Dis..

[24] Rachii D., Bezold E. L., Wuest W. M., Minbiole K. P. C. (2025). Bushy-Tailed
Multicationic Quaternary Phosphonium Compounds: Potent Amphiphilic
Disinfectants with Promising Therapeutic Indices. ChemMedChem.

[25] Leatherbury M. S., Thierer L. M., Sanchez C. A., Vargas-Cuebas G. G., Petersen A. A., Amoo L. E., Bezold E. L., Washington K. C., Mistrot M. B., Zdilla M. J., Wuest W. M., Minbiole K. P. C. (2024). Chimeric
Amphiphilic Disinfectants: Quaternary Ammonium/Quaternary Phosphonium
Hybrid Structures. ChemMedChem.

[26] Sanchez C. A., Vargas-Cuebas G. G., Michaud M. E., Allen R. A., Morrison-Lewis K. R., Siddiqui S., Minbiole K. P. C., Wuest W. M. (2024). Highly Effective
Biocides against Pseudomonas aeruginosa Reveal New Mechanistic Insights Across Gram-Negative Bacteria. ACS Infect. Dis..

[27] François G., François G., Patrick P., Marion A., Racha B., Richard B., Vincent C., Jean-Christophe G. (2019). The Transcriptional
Repressor SmvR Is Important for Decreased Chlorhexidine Susceptibility
in Enterobacter Cloacae Complex. Antimicrob.
Agents Chemother..

[28] Asante J. Y. D., Casey C. M., Bezold E. L., Fernando A., McDonough D., Wuest W. M., Minbiole K. P. C. (2025). Resorcinol-Based Bolaamphiphilic
Quaternary Ammonium Compounds. ChemMedChem.

[29] Mondal P., Roy S., Dey J., Dasgupta S. B. (2024). Impact of Linker Groups on Self-Assembly,
Gene Transfection, Antibacterial Activity, and In Vitro Cytotoxicity
of Cationic Bolaamphiphiles. ACS Appl. Bio Mater..

[30] Ljungquist O., Magda M., Giske C. G., Tellapragada C., Nazarchuk O., Dmytriiev D., Thofte O., Öhnström V., Matuschek E., Blom A. M., Riesbeck K. (2024). Pandrug-Resistant Klebsiella pneumoniae Isolated from Ukrainian War
Victims Are Hypervirulent. J. Infect..

[31] Mc
Gann P. T., Lebreton F., Jones B. T., Dao H. D., Martin M. J., Nelson M. J., Luo T., Wyatt A. C., Smedberg J. R., Kettlewell J. M., Cohee B. M., Hawley-Molloy J. S., Bennett J. W. (2023). Six Extensively Drug-Resistant Bacteria in an Injured
Soldier, Ukraine. Emerging Infect. Dis..

[32] Kücken D., Feucht H.-H., Kaulfers P.-M. (2000). Association of QacE and QacE Δ1
with Multiple Resistance to Antibiotics and Antiseptics in Clinical
Isolates of Gram-Negative Bacteria. FEMS Microbiol.
Lett..

[33] Kazama H., Hamashima H., Sasatsu M., Arai T. (1999). Characterization of
the Antiseptic-Resistance Gene QacEΔ1 Isolated from Clinical
and Environmental Isolates of Vibrio Parahaemolyticus and Vibrio Cholerae
Non-O1. FEMS Microbiol. Lett..

[34] Forbes C. C., DiVittorio K. M., Smith B. D. (2006). Bolaamphiphiles Promote Phospholipid
Translocation Across Vesicle Membranes. J. Am.
Chem. Soc..

[35] Piñeiro L., Novo M., Al-Soufi W. (2015). Fluorescence Emission of Pyrene in
Surfactant Solutions. Adv. Colloid Interface
Sci..

[36] Perinelli D. R., Cespi M., Lorusso N., Palmieri G. F., Bonacucina G., Blasi P. (2020). Surfactant Self-Assembling and Critical Micelle Concentration: One
Approach Fits All?. Langmuir.

[37] Inácio Â.
S., Mesquita K. A., Baptista M., Ramalho-Santos J., Vaz W. L. C., Vieira O. V. (2011). In Vitro
Surfactant Structure-Toxicity
Relationships: Implications for Surfactant Use in Sexually Transmitted
Infection Prophylaxis and Contraception. PLoS
One.

[38] Regen S. L. (2021). Membrane-Disrupting
Molecules as Therapeutic Agents: A Cautionary Note. JACS Au.

[39] Perinelli D. R., Cespi M., Casettari L., Vllasaliu D., Cangiotti M., Ottaviani M. F., Giorgioni G., Bonacucina G., Palmieri G. F. (2016). Correlation among Chemical Structure,
Surface Properties and Cytotoxicity of N-Acyl Alanine and Serine Surfactants. Eur. J. Pharm. Biopharm..

[40] Vargas-Cuebas G. G., Sanchez C. A., Brayton S. R., Nikoloff A., Masters R., Minbiole K. P. C., Wuest W. M. (2024). Exploring
the Correlation of Dynamic
Surface Tension with Antimicrobial Activities of Quaternary Ammonium-Based
Disinfectants. ChemMedChem.

[41] Li H., Hu D., Liang F., Huang X., Zhu Q. (2020). Influence Factors on
the Critical Micelle Concentration Determination Using Pyrene as a
Probe and a Simple Method of Preparing Samples. R. Soc. Open Sci..

[42] Zeng P., Zhang G., Rao A., Bowles W., Wiedmann T. S. (2009). Concentration
Dependent Aggregation Properties of Chlorhexidine Salts. Int. J. Pharm..

[43] Muthuraman G., Chandrasekara Pillai K., Moon I.-S. (2019). Electrochemical Analysis of Aqueous
Benzalkonium Chloride Micellar Solution and Its Mediated Electrocatalytic
De-Chlorination Application. Catalysts.

[44] Han Z., Yang X., Liu Y. (2015). Micellar and
Interfacial Behavior
of Mixed Systems Containing Glycoside-Based Surfactant and Cationic
Didecyldimethylammonium Chloride. J. Surfactants
Deterg..

[45] Kim M.-C., Nam S. S., Lee C., Park J. S., Yoo H., Jin H. M., Sim E., Lee S.-Y. (2025). Length of Alkyl
Chain Spacer Modulates Aggregation Behavior and Coordination Capability
of Histidyl Bolaamphiphiles. Mater. Today Chem..

