# Supplementary material for: Bisphosphonium Amphiphiles Yield Insights into Gram-Negative Bacterial Disinfectant Resistance and Cell Membrane Interactions
Source: ACS Omega. 2025 Jun 5;10(23):25076–83. doi: 10.1021/acsomega.5c03308 (PMC12177629; doi:10.1021/acsomega.5c03308)
Supplement: Supplementary file 1 [file ao5c03308_si_001.pdf]

Supporting Information for Publication

Bisphosphonium amphiphiles yield insights into gram-negative bacterial disinfectant resistance and cell membrane interactions

Elise L. Bezold[a†], Abigail L. E. Young[a†], Carson J. Jaworski[b], Kevin P.C. Minbiole[c], Christian A. Sanchez[b*], and William M. Wuest[a*]

[a]*Department of Chemistry, Emory University, Atlanta, GA 30322*
[c]*Department of Chemistry and Biochemistry, Samford University, Birmingham, AL 35229*
[d]*Department of Chemistry and Biochemistry, Villanova University, Villanova, PA 19085*
[†] *Authors contributed equally*

Christian A. Sanchez: *csanche1@samford.edu*

William M. Wuest: *wwuest@emory.edu*

Table of Contents

I. Synthetic Procedures
II. Bacterial Strains
III. Biological Assays
IV. Pyrene critical micelle determination data
V. Characterization
VI. References

I. Synthesis

Preparation of **P2P-10,10**, **P3P-10,10**, **P4P-10,10**, **P5P-10,10**, and **P6P-10,10** were synthesized based on previous reports.[1]

Preparation of **P8P-10,10**

To 1,8-bis(diphenylphosphino)octane (0.100 g, 0.207 mmol, 1 eq) was added 1-bromodecane (109 µL, 0.117 g, 0.518 mmol, 2.5 eq) and DMF (3 mL). The solution was heated to 100ºC and stirred for 24 hours. After cooling to room temperature, the contents of the reaction flask were concentrated using rotary evaporation. The resulting oil was triturated with 1:1 ether:hexanes (20 mL) and cooled at -25 °C overnight. The trituration solvent was discarded, and the resulting precipitate was dissolved in dichloromethane (5 mL). The solution was transferred to a clean vial and concentrated using rotary evaporation to afford P8P-10,10 as a white crystalline powder (0.168 mg, 88%); **^{1}H NMR** (400 MHz, $CDCl_3$) δ 7.91 (dd, *J* = 12.0, 7.7 Hz, 8H), 7.77 (dd, *J* = 8.3, 6.4 Hz, 4H), 7.69 (td, *J* = 7.6, 3.1 Hz, 8H), 3.31 (d, *J* = 13.6 Hz, 8H), 1.48 (s, 16H), 1.31 – 1.22 (m, 11H), 1.18 (s, 17H), 0.85 (t, *J* = 6.9 Hz, 6H). **^{13}C NMR** (101 MHz, $CDCl_3$) δ 134.74, 134.71, 133.44, 133.34, 130.48, 130.36, 118.54, 117.72, 77.36, 31.93, 30.64, 30.49, 29.80, 29.64, 29.55, 29.35, 29.20, 27.73, 22.75, 22.37, 22.31, 22.10, 21.89, 21.75, 21.70, 21.61, 14.22. **^{31}P NMR** (162 MHz, $CDCl_3$) δ 28.58. **HRMS** Accurate Mass (ESI+): Found 382.7773, C52H78P2[-2Br-]2+ requires 382.2789.

II. Bacterial Strains and Growth Conditions

Pseudomonas aeruginosa PAO1 was acquired from Prof. Bettina A. Buttaro (Lewis Katz School of Medicine, Temple University). MRSN 122268, 740699, 740761, 122323, 122357, 567268, 122372, 821382 are clinical isolates of *P. aeruginosa* obtained from the Multi-drug resistant Organism Repository and Surveillance Network (MRSN) provided by the Department of Defense. Bacterial strains were streaked onto lysogeny broth (LB) agar plates and incubated at 37°C overnight. Single colonies were used to inoculate liquid cultures and incubated for 18 h at 37°C with shaking.

III. Biological Assays

Minimum Inhibitory Concentration (MIC) Assays

To determine the MIC values, compounds were serially diluted two-fold from stock solutions (1.0 mM) to yield twelve 100 μL test concentrations, wherein the starting concentration of DMSO was 2.5%. Overnight cultures of each strain were diluted to ca. 10^6 CFU/mL in MHB and regrown to mid-exponential phase, as determined by optical density recorded at 600 nm (OD_{600}). All cultures were then diluted again to ca. 10^6 CFU/mL and 100 μL were inoculated into each well of a U-bottom 96-well plate (Falcon, 351177) containing 100 μL of compound solution. Plates were incubated statically at 37 ºC for 72 hours upon which wells were evaluated visually for bacterial growth. The MIC was determined as the lowest concentration of compound resulting in no bacterial growth visible to the naked eye, based on the highest value in three independent experiments. Aqueous DMSO controls were conducted for each strain. All *P. aeruginosa* strains were grown with shaking at 37 °C overnight from freezer stocks in 5 mL of BD Mueller–Hinton broth (MHB).

Red Blood Cell (RBC) Lysis Assay ($Lysis_{20}$)

RBC lysis assays were performed on mechanically defibrinated sheep blood (Hemostat Labs: DSB030). An aliquot of 1.5 mL blood was placed into a microcentrifuge tube and centrifuged at 10,000 rpm for ten min. The supernatant was removed, and the cells were resuspended with 1 mL of phosphate-buffered saline (PBS). The suspension was centrifuged as described above, the supernatant was removed, and cells were resuspended 4 additional times in 1 mL PBS. The final cell suspension was diluted twenty-fold with PBS. Compounds were serially diluted with PBS two-fold from stock solutions (1.0 mM) to yield 100 μL of twelve test concentrations on a U-bottom 96-well plate (Falcon, 351177), wherein the starting concentration of DMSO was 2.5%. To each of the wells, 100 μL of the twenty-fold suspension dilution was then inoculated. The concentration of DMSO in the first well was 2.5%, resulting in DMSO-induced lysis at all concentrations >63 μM. TritonX (1% by volume) served as a positive control (100% lysis marker) and sterile PBS served as a negative control (0% lysis marker). Samples were

then placed in an incubator at 37 ºC and shaken at 200 rpm. After 1 hour, the samples were centrifuged at 2,000 rpm for ten minutes. 100 µL of the supernatant was transferred to a fresh flat-bottom 96-well plate (Corning, 351172), and the absorbance of the supernatant was measured with a UV spectrometer at a 540 nm wavelength. The concentration inducing 20% RBC lysis was then calculated for each compound based upon the absorbances of the TritonX and PBS controls. Aqueous DMSO controls were conducted as appropriate for each compound.

NPN Uptake Assay

P. aeruginosa PAO1 were grown overnight in LB, then regrown from a 1:100 dilution in fresh media for 5 hours to an OD600 of 0.500. Cells were harvested by centrifugation (4000 rpm, 25ºC, 10 min), washed twice with assay buffer (5 mM HEPES, 5 mM glucose, pH 7.2), and resuspended in assay buffer to a final OD600 of 1. Then, 100 µL of washed cells and 100 µL of assay buffer containing 20 µM NPN were together and incubated for 10-30 min. 198 µl of cells and NPN added to a 96-well optical-bottom black plate. Either 2 µL of a chemical compound or the corresponding solvent was added to each well, and fluorescence was immediately monitored at an excitation wavelength of 350 nm and an emission wavelength of 420 nm for 7 minutes at 30 second intervals.

F_{obs} = NPN + cells + compound

$F_{control}$ = NPN + cells

F_b = NPN

NPN uptake = (F_{obs} – F_B)—($F_{control}$-F_B)

20 µM NPN in assay buffer was made from a 5 mM stock of NPN in acetone.

$DISC_3$-(5) Depolarization Assay

P. aeruginosa PAO1 were grown overnight in LB, then regrown from a 1:100 dilution in fresh media. Midlog phase bacteria (OD_{600} = 0.4 – 0.6) were harvested, washed once, and resuspended in HEPES buffer (5 mM HEPES at pH 7.2) to an optical absorbance of OD_{600} = 0.05. Then, 100 µL of 10 mM EDTA was added to 5 mL of resuspended cells for a final concentration of 200 µM EDTA. The bacterial solution was then gently mixed and then let sit for 2 minutes. Afterwards, 5 µL of 0.75 mM $DISC_3$(5) was added to the solution for a final concentration of 0.75 µM. Following another gentle mix, the solution was left to incubate in the dark at 37ºC. After incubation, 125 µL of 4M KCl was added to the cells for a final concentration of 100 mM KCl. Finally, 198 µL of cells and $DISC_3$(5) added to a 96-well optical-bottom black plate. Either 2 µL of a chemical compound, or the

corresponding solvent, was added to each well. The excitation wavelength was 622 nm, and the emission wavelength was 670 nm. The release of $DiSC_3(5)$ was measured by the increase in fluorescence of $DiSC_3(5)$ for 60 min as a measure of inner membrane depolarization.

Pyrene CMC determination assay

10.1 mg of pyrene and 80 mL of ethanol were added into a 100 mL volumetric flask. After shaking well until fully dissolved, ethanol was filled to the mark to prepare a 500 μM solution. A 1:1000 dilution of the pyrene solution in water was performed to prepare a 0.5 μM solution of pyrene. A 1.2 mM stock solution of each P(n)P-10,10 compound was prepared with the pyrene solution. This was the test solution used for fluorescence experiments, containing both 0.5 μM pyrene and 1.2 mM of surfactant.

The test solution was further diluted in a 96-well plate with additional 0.5 μM pyrene solution to obtain surfactant concentrations between 0.5 mM and 1.2 mM, while maintaining 0.5 μM of pyrene. The final volume in each well was 200 μL. Exciting at 336 nm, the fluorescence emission of pyrene at 374 nm (I1) and 382 nm (I3) was recorded. The I1/I3 intensity ratio was plotted against the concentration of each sample. The CMC value was determined from the intersection of the best-fit lines, corresponding to the minimum surfactant concentration required for the formation of stable micelles in the aqueous solution.

IV. Pyrene critical micelle concentration data

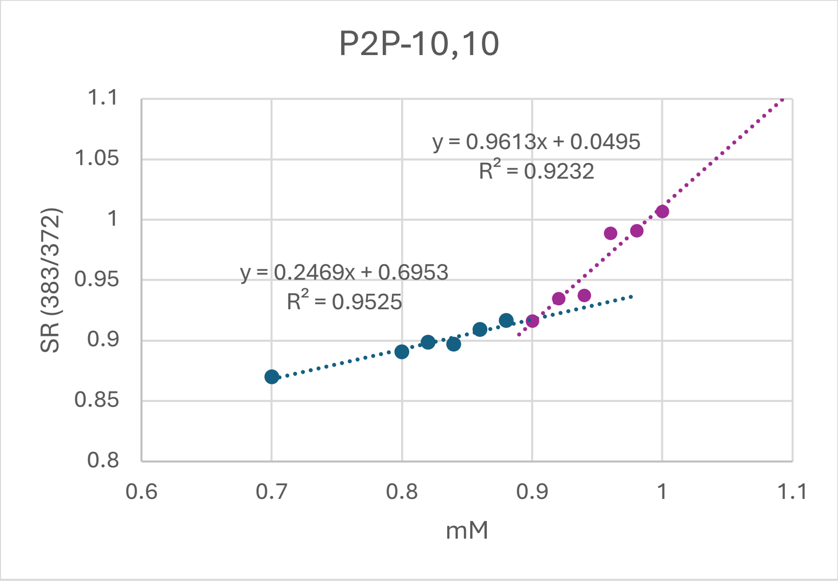

x	y
0.6	0.86
0.7	0.87
0.8	0.89
0.82	0.90
0.84	0.90
0.86	0.91
0.88	0.92
0.9	0.92
0.92	0.94
0.94	0.94
0.96	0.99
0.98	0.99
1	1.01

Intersection of best fit lines at 0.898 mM

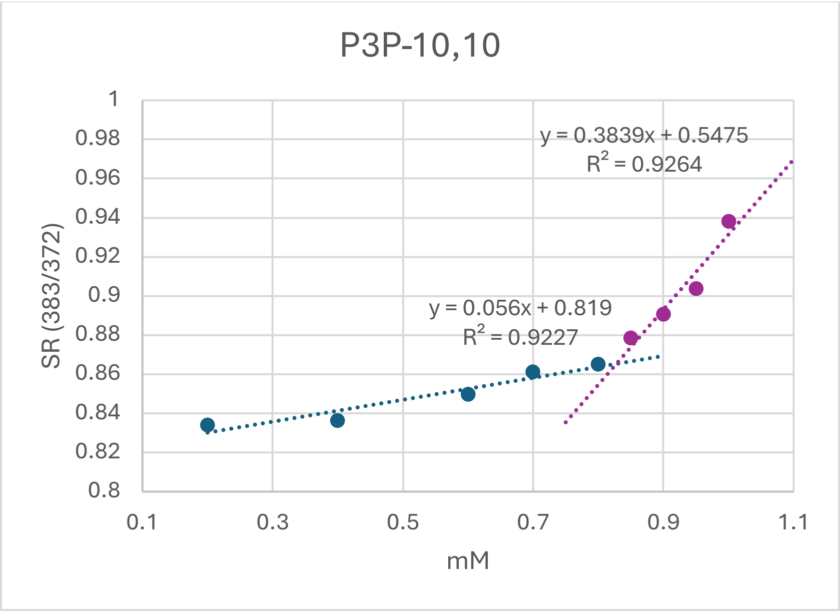

x	y
1	0.94
0.95	0.90
0.9	0.89
0.85	0.88
0.8	0.86
0.7	0.86
0.6	0.85
0.4	0.84
0.2	0.83
0.05	0.85

Intersection of best fit lines at 0.828 mM

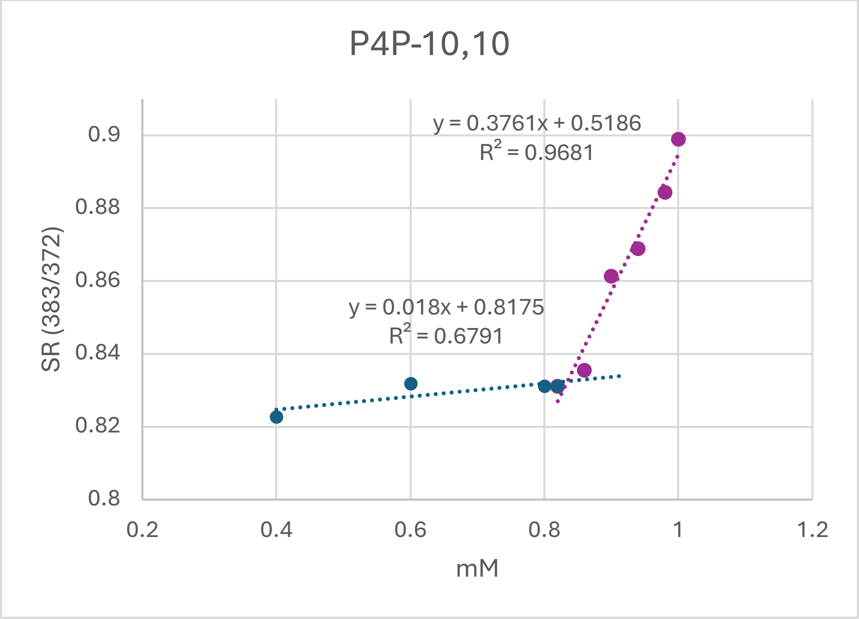

x	y
1	0.90
0.98	0.88
0.94	0.87
0.9	0.86
0.86	0.84
0.82	0.83
0.8	0.83
0.6	0.83
0.4	0.82

Intersection of best fit lines at 0.834 mM

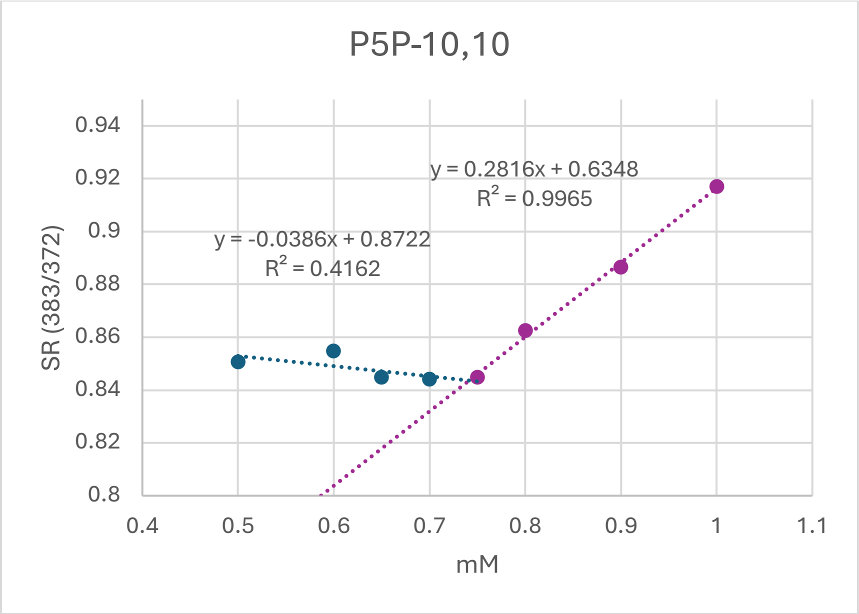

x	y
1	0.92
0.9	0.89
0.8	0.86
0.75	0.84
0.7	0.84
0.65	0.84
0.6	0.85
0.5	0.85

Intersection of best fit lines at 0.741 mM

V. Characterization

P8P-10,10

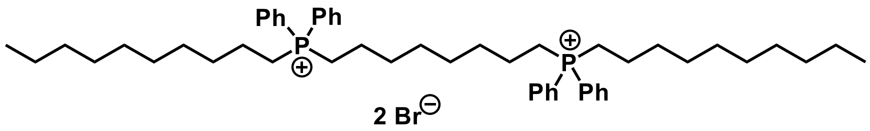

^{1}H NMR

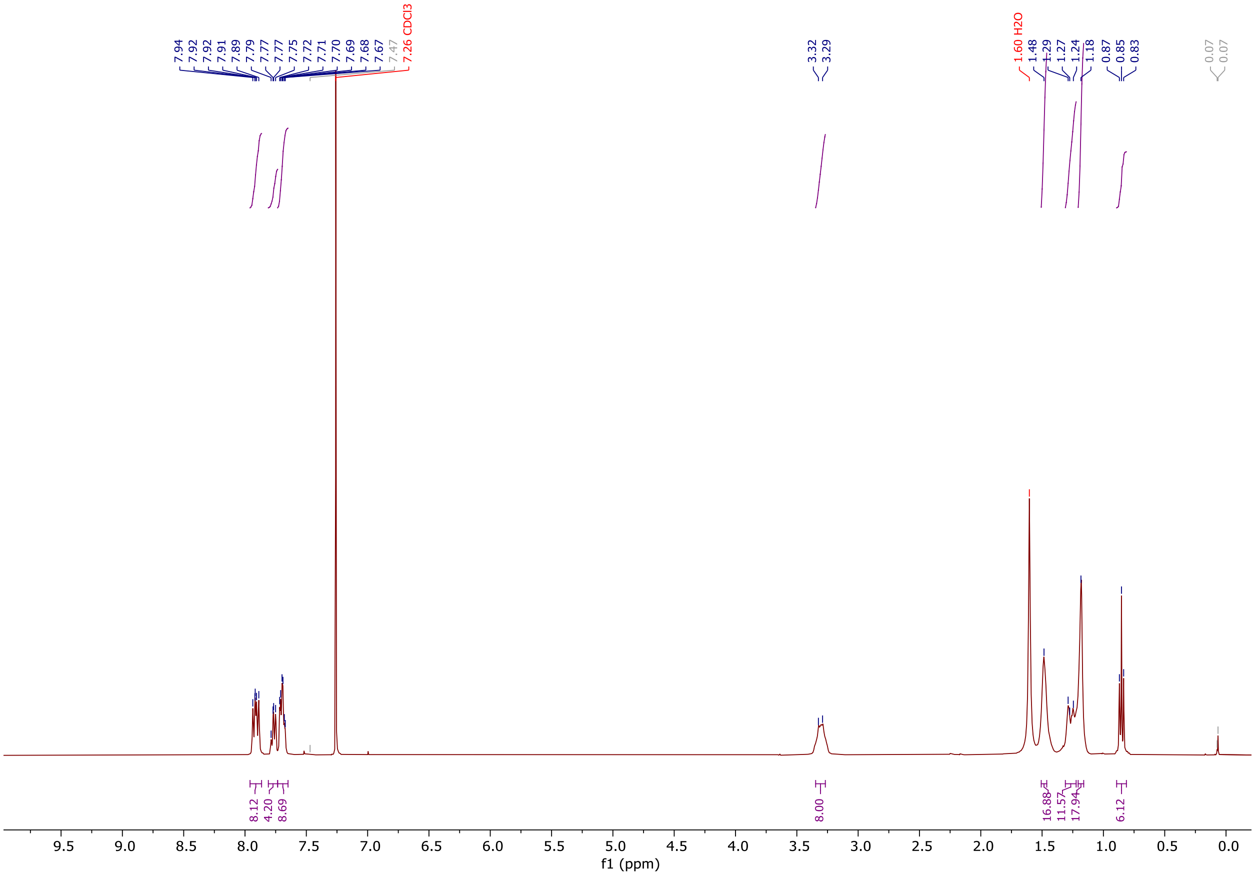

^{1}H NMR (400 MHz, $CDCl_3$) δ 7.91 (dd, J = 12.0, 7.7 Hz, 8H), 7.77 (dd, J = 8.3, 6.4 Hz, 4H), 7.69 (td, J = 7.6, 3.1 Hz, 8H), 3.31 (d, J = 13.6 Hz, 8H), 1.48 (s, 16H), 1.31 – 1.22 (m, 11H), 1.18 (s, 17H), 0.85 (t, J = 6.9 Hz, 6H).

P8P-10,10

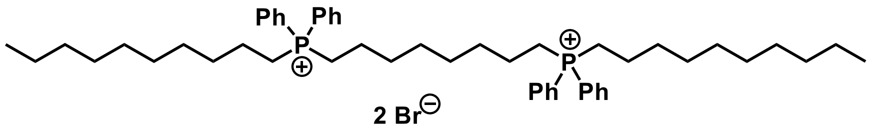

^{31}P NMR:

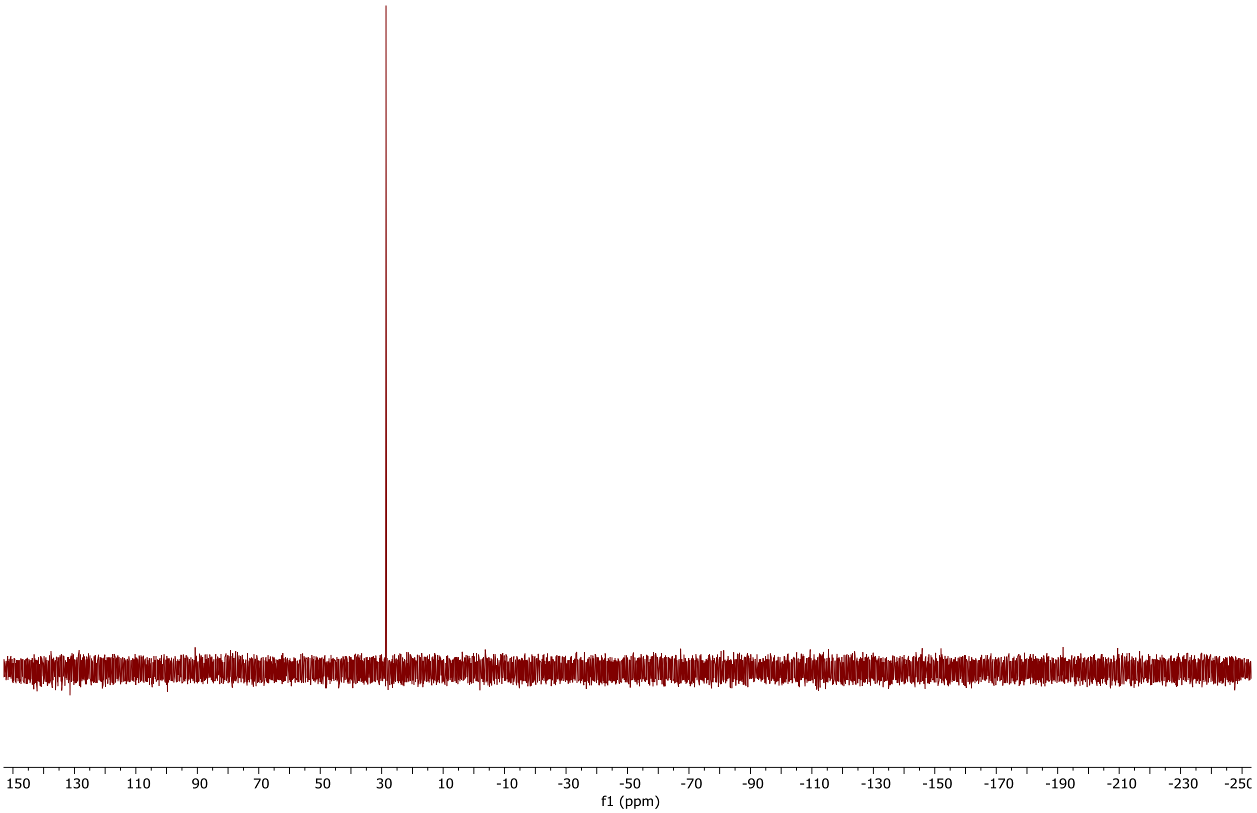

^{31}P NMR (162 MHz, $CDCl_3$) δ 28.58.

P8P-10,10

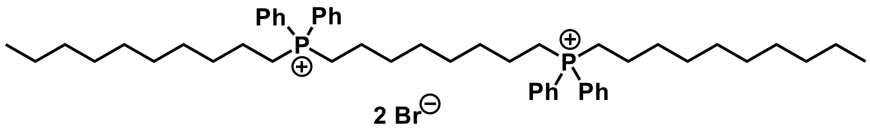

^{13}C NMR:

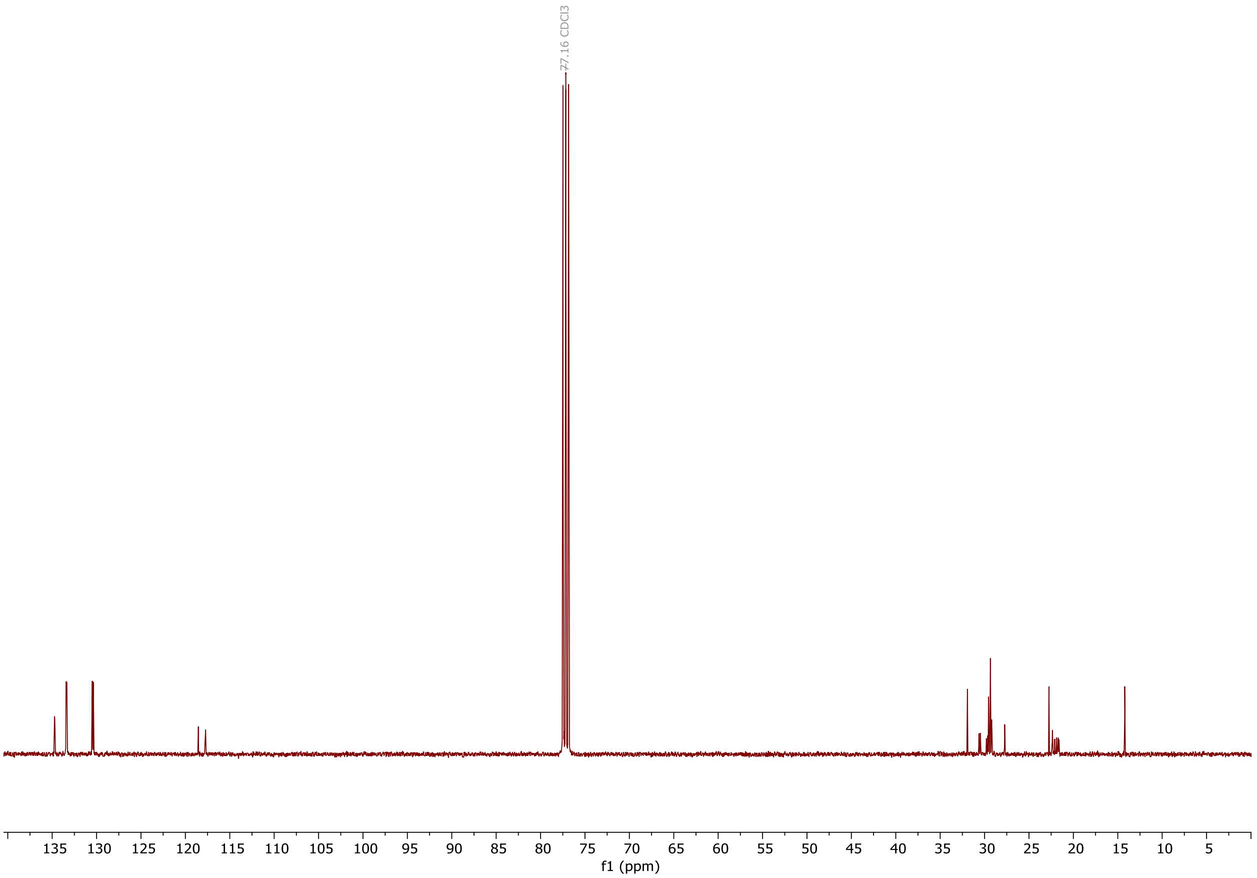

^{13}C NMR (101 MHz, $CDCl_3$) δ 134.74, 134.71, 133.44, 133.34, 130.48, 130.36, 118.54, 117.72, 77.36, 31.93, 30.64, 30.49, 29.80, 29.64, 29.55, 29.35, 29.20, 27.73, 22.75, 22.37, 22.31, 22.10, 21.89, 21.75, 21.70, 21.61, 14.22.

VI. References

1. Sommer, K.J.; Michaud, M.E.;I Hogue, C.E.; Scharnow, A.M.; Amoo, L.E.; Petersen, A.A.; Carden, R.G.; Minbiole, K.P.C.; Wuest, W.M. Quaternary Phosphonium Compounds: An Examination of Non-Nitrogenous Cationic Amphiphiles That Evade Disinfectant Resistance. *ACS Infect. Dis.* **2022**, *8* (2), 387-397.
